# A System of Practical Medicine

**Published:** 1885-10

**Authors:** 


**A System of Practical Medicine,** by American authors. Edited by Wm. Pepper, M.D., LL.D., Provost and Professor of the Theory and Practice of Medicine, and of Clinical Medicine, in the University of Pennsylvania, assisted by Louis Starr, M.D., Clinical Professor of Diseases of Children in the Hospital of the University of Pennsylvania. Volume II. General Diseases, and Diseases of the Digestive System. Philadelphia: Lea Brothers & Co. 1885.

In noticing the first volume of this great work, we stated that if the succeeding volumes were equal to the first, the complete work would be, indeed, a noble addition to American Medicine. All those who have subscribed to the work will, we are confident, be entirely satisfied with the contents of this volume. We now venture the prediction that, for real practical value to the American physician, this great work will far surpass any other "System of Medicine" heretofore published. We hope that all of our readers will be numbered among its subscribers, and we are confident that they will consider the money expended upon it as a most profitable investment. The portion of the work devoted to general diseases is in this volume continued and completed. Prof. Howard, of Montreal, gives a most admirable treatise on Rheumatism, while Dr. Draper, of New York, contributes an article on Gout. Rachitis, Scurvy Purpural, Diabetes Mellitus, Scrofula and Hereditary Syphilis are respectively considered by Jacobi, Wales, Atkinson Tyson, Lynch, and J. William White. The Diseases of the Digestive System follow next in order, and occupy the remainder of a volume of nearly 1,400 pages. The Diseases of the Mouth, Tongue, Tonsils, Pharynx and Œsophagus receive masterly treatment from the pen of J. Solis Cohen. The various affections of the Stomach, Intestinal Indigestion, Constipation, and other affections of the bowels, receive careful and exact attention by various distinguished writers. Bartholow presents an admirable chapter on Diseases of the Liver, and Alonzo Clark on Peritonitis. Our space will not allow us to give the names of authors or titles in full of all the articles, much less to consider their merits individually. It is sufficient to say that this volume, as a whole, affords little opportunity for other than favorable criticism. We congratulate the distinguished editor and the American profession upon the great success and completeness of the first two volumes issued.

